# Prospective Evaluation of the Concordance of Commercial Circulating Tumor DNA Alterations with Tumor-Based Sequencing across Multiple Soft Tissue Sarcoma Subtypes

**DOI:** 10.3390/cancers11121829

**Published:** 2019-11-21

**Authors:** Bryce Demoret, Jeff Gregg, David A. Liebner, Gabriel Tinoco, Scott Lenobel, James L. Chen

**Affiliations:** 1 Division of Medical Oncology, The Ohio State University, Columbus, OH 43210, USA; BD550418@ohio.edu (B.D.); David.Liebner@osumc.edu (D.A.L.); Gabriel.Tinoco@osumc.edu (G.T.); 2 Department of Pathology and Laboratory Medicine, UC Davis Health and Foundation Medicine Inc., Cambridge, MA 02141, USA; jgregg@foundationmedicine.com; 3 Department of Biomedical Informatics, The Ohio State University, Columbus, OH 43210, USA; 4 Department of Radiology, The Ohio State University, Columbus, OH 43210, USA; Scott.Lenobel@osumc.edu

**Keywords:** Circulating tumor DNA, liquid biopsy, soft tissue sarcoma

## Abstract

Soft tissue sarcomas (STS) are diverse tumors with heterogenous alterations. Platforms to detect circulating tumor DNA (ctDNA) have rapidly increased in popularity as they may avoid invasive biopsy morbidity. However, ctDNA profiling concordance with standard solid tumor comprehensive genomic profiling (CGP) is poorly characterized. Here, we report the outcomes of a single-institution experience comparing mutational results from commercial ctDNA and solid tumor CGP in advanced STS subjects. We identified STS subjects who had undergone solid tumor based CGP in four distinct cohorts: Dedifferentiated liposarcoma (DDLPS), leiomyosarcoma (LMS), undifferentiated pleomorphic sarcoma (UPS), and gastrointestinal stromal tumor (GIST). Subjects with radiographically measurable tumor were profiled using a commercial ctDNA CGP panel. Overlapping genes/exons on both biopsy panels were analyzed. Twenty-four subjects completed both ctDNA and solid tumor CGP. ctDNA was detected in 18/24 subjects. Subject level concordance rates in all overlapping genes were: LMS = 4/6; UPS = 2/6; DDLPS = 1/6; GIST = 0/6. Copy number alterations were notably poorly concordant. For subjects with short variant alterations and detectable tumor fractions, concordance with solid tumor CGP was 76% (13/17). LMS subjects had the highest median tumor fraction and concordance. No correlation was seen between tumor fraction or radiographic tumor volume largely driven by low estimated tumor fraction. A limitation of the study is that only targeted sequencing was performed. However, given the poor concordance in commonly altered genes, ctDNA panels in sarcoma cannot be broadly applied. Further, more extensive studies will need to be performed.

## 1. Introduction

Soft tissue sarcomas (STS) represent a diverse population of tumors of mesenchymal origin. This heterogeneity in STS is driven by multiple molecular pathogenesis paths which span the gamut from fusions, copy number alterations (CNA), to point mutations in key genes. The process of DNA/RNA sequencing and outcome reporting of known oncogenic areas of the cancer genome is sometimes termed comprehensive genomic profiling (CGP). The advent and routine use of CGP platforms provide critical insight not only for treatment selection but also for upfront diagnosis in soft tissue sarcomas [1,2,3]. Despite the potential utility of routine CGP use in STS, CGP is often stymied by the availability of obtainable biopsy specimens. STS locations are often in or adjacent to critical structures which may result in a morbid procedure. Furthermore, serial biopsies reliant on tumor tissue for monitoring of the sarcoma genome can be a morbid procedure. Thus, other CGP testing modalities are desired.

To this end, advances in DNA sequencing and bioinformatics platforms have permitted the characterization and quantification of alterations in circulating tumor DNA (ctDNA). ctDNA can be derived from bodily fluids, most commonly blood, and thus has increased in popularity due to its ease of collection [4]. ctDNA liquid biopsies in solid tumors such as lung and breast may provide actionable information including real-time, holistic tumor pathogenesis, tumor progression and prognosis, and early-stage detection [2,5,6]. Yet the utility of ctDNA in sarcoma is not well-characterized.

Prior reports have been hindered by restricted subject sizes and variable interstudy methods have made it difficult to draw firm conclusions. They also have been performed using research grade platforms. In the largest study of its time, a 2018 investigation (Eastley et al. [7]) demonstrated in 11 STS subjects that ctDNA was detectable in some subjects. Since then, few additional efforts have been reported. A more recent study specifically looked at 32 leiomyosarcoma (LMS) subjects for detectable ctDNA associated with tumor size and disease progression [8]. Their results suggest LMS ctDNA levels are associated with disease burden and could further be used as an indicator of disease progression or response to systemic therapy. Others have demonstrated that circulating fusions are detectable in Ewing and myxoid liposarcomas subjects using digital droplet-based PCR [9].

To address the utility of ctDNA in STS, we prospectively evaluated a commercial, CLIA-certified ctDNA panel in soft tissue sarcoma types and compared the results to solid tumor CGP from matched subjects. We prospectively enrolled and analyzed STS subjects with measurable disease using tumor profiling (FoundationOne^®^ Heme, (F1Heme)) and a circulating ctDNA panel (FoundationACT™, (F1ACT)) (Foundation Medicine, Cambridge, MA, USA) [10,11]. Our results highlight notable heterogeneity among sarcoma subtypes with most releasing minimal ctDNA.

## 2. Results

### 2.1. Overview Report and Analysis Workflow

**Figure 1:**
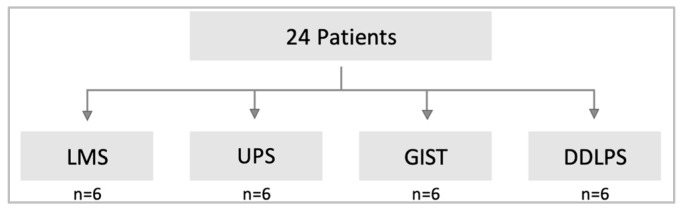
Schema of subject grouping. 24 subjects were dived into four cohorts based on diagnosis namely: leiomyosarcoma (LMS), undifferentiated pleomorphic sarcoma (UPS), gastrointestinal stromal tumor (GIST), and dedifferentiated liposarcoma (DDLPS). Circulating tumor DNA (ctDNA) were detectable in blood as follows: 6/6 LMS, 5/6 UPS, 4/6 GIST, 3/6 DDLPS.

From June 2016 to November 2018, 24 subjects with advanced stage metastatic STS were enrolled into four distinct cohorts in this study. Subjects had an average age of 67 and were 54% female (Table 1). Six subjects were in each of the following cohorts: dedifferentiated liposarcoma (DDLPS), leiomyosarcoma (LMS), myxofibrohistiocytic or undifferentiated pleomorphic sarcoma (UPS), or gastrointestinal stromal tumor (GIST) (Figure 1). As part of entry criteria, each subject had previously successfully undergone a solid tumor CGP (F1Heme). Upon confirmation of radiographically measurable disease, a blood sample was collected for evaluation using a ctDNA liquid panel (F1ACT) which evaluates select substitutions, indels, copy number amplifications, and rearrangements as detailed in Methods [12]. All analyzed data is available in the Supplemental Worksheet provided.

### 2.2. Circulating Tumor Fractions Are Low in STS Subjects and Poorly Correlate with Radiographically Measured Tumor Volumes

**Figure 2:**
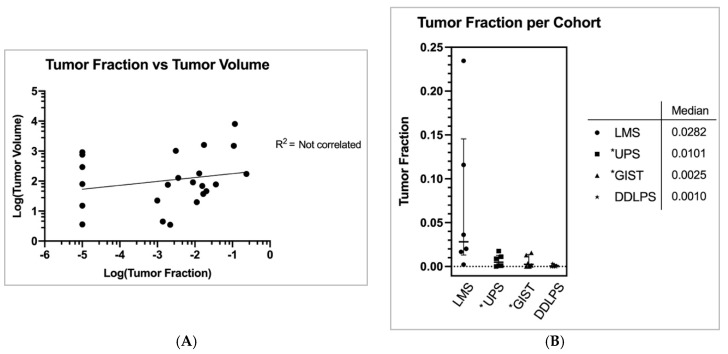
(**A**) Estimated tumor fraction, i.e., the fraction cell free DNA derived from tumor rather than non-cancerous tissue, was calculated per Foundation Medicine. Each subject was administered an estimated tumor fraction (TF) seen here in this figure. The median TF in each cohort roughly correlates with the observed concordances from Figure 3B. Figure graphically depicts median with interquartile range. * = TF reevaluated due to the presence of germline mutations. (**B**) Each subject’s volumetric sum of all cancerous lesions were added and plotted against Foundation Medicine’s computed estimated tumor fraction. A logarithmic scale was applied to both axes for better visual representation. Subjects with TF of zero were provided with an arbitrary TF of 0.00001 for the log-log plot. No correlation was observed. Moreover, no significant correlation was observed when comparing TF vs number of metastases nor tumor volume vs number of metastases (Figure S1A,B).

Of 24 STS subjects evaluated, 18 (75%) subjects had detectable ctDNA for further analysis. For simplicity, here we define tumor fraction (TF) as the total amount of ctDNA/non-tumor cell-free DNA (cfDNA) [2]. Full calculation methodology is characterized in the Methods. The mean coverage for the ctDNA assays was 9576 and median coverage was 11,026 (min coverage was 4497 and max coverage was 14,935). STS overall mean and median tumor fraction was 2.6% and 0.36%, respectively (std. dev.: 0.055, range 0.0% to 23.5%). LMS subjects had the highest median TF of 2.8% compared to UPS = 1.0%, GIST = 0.25%, and DDLPS = 0.10% (Figure 2A) (std. devs.: 0.09, 0.007, 0.007, 0.001, respectively).

Each subject had CT or MRI imaging that was performed within one month of blood collection for ctDNA evaluation. A single board-certified radiologist quantitated total tumor volumes for all subjects. We evaluated each subjects’ total tumor volume (combining all metastatic lesions) at the time of F1ACT collection and compared the results with subjects’ associated tumor fraction (Table S1). Tumor volumes across the four subtypes were comparable (Figure S1C). Tumor volume was variable based on patient and subtype (mean = 618.1 cm^3^. std. dev.: 1686.5, range 3.5 to 8069). There was no clear correlation between subjects’ TF and tumor volume (R^2^ = NS) (Figure 2B). Neither was there correlation between the number of metastatic sites and TF (R^2^ = NS, Figure S1A,B).

### 2.3. Concordance between Solid Tumor and Blood-Based Sequencing Is Driven Primarily by STS Subtype

**Figure 3:**
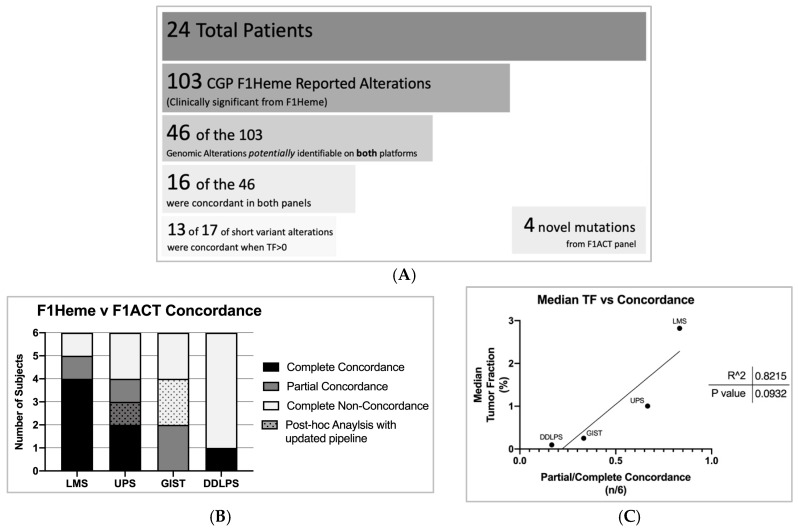
(**A**) Twenty-four subjects who underwent F1Heme profiling identified a total of 103 genomic alterations. Of the 103 alterations in F1Heme, the F1ACT assay had the power to detect 46 of those alterations. F1ACT reported 16/46 mutations concordant with F1Heme and identified four additional mutations. Six soft tissue sarcomas (STS) subjects failed to report any quantifiable ctDNA estimated TF. For short variants with TF > 0, 13 of 17 alterations were concordant in the liquid/solid CGP. (**B**) Twenty-four subjects (LMS *n* = 6, UPS *n* = 6, GIST *n* = 6, DDLPS *n* = 6) tumor mutations concordances between F1Heme and F1ACT assays per STS subtype. 7/24 subjects acquired complete concordance across tests, 5/24 were partially concordant (at least one—but not all—mutations identified), and 12/24 subjects failed to identify any original comprehensive genomic profiling (CGP) mutations and were completely non-concordant. LMS subjects harbored the highest concordance. After a software pipeline update that improved the performance of F1ACT’s detection capabilities one UPS subject became completely concordant and two GIST subjects went from completely non-concordant to partially concordant post identifying at least one CGP reported mutation. (**C**) Median estimated TF compared to the fraction of subjects which were either partially or completely concordant per STS subtype.

The solid tumor sequencing panel resulted in a total of 103 alterations (LMS *n* = 22, DDLPS *n* = 33, GIST *n* = 18, UPS *n* = 30) and the ctDNA panel (F1ACT) reported 20 alterations (LMS *n* = 7, DDLPS *n* = 5, GIST *n* = 2, UPS *n* = 6). It is important to note that of the 103 genomic mutations identified in F1Heme only 46 of the 103 alterations overlapped with F1ACT’s panel for identification. Therefore, 57 of the 103 alterations, including frequently STS altered genes such as RB1, ATRX, and FRS2, were not interrogated on F1ACT’s panel. F1ACT was concordant with 16 of the 46 mutations (Figure 3A). For short variants in cases with TF > 0, 13 of 17 alterations were detected. F1ACT reported four novel mutations not identified in F1Heme (Figure S2).

To evaluate the effect of STS subtype on the concordance between sequencing platforms we classified subjects as being completely concordant, partially concordant (at least one but not all mutations identified), and non-concordant within each subtype. Looking only at potentially identifiable mutations, a total of 7 of 24 subjects registered as completely concordant, 5 of 24 partially concordant, and 12 of 24 completely non-concordant (Figure 3B). After this study was completed, the F1ACT pipeline was updated with improved performance. To evaluate whether the new pipeline improved variant calling in this data set, we performed a post-hoc analysis of the data set with the new pipeline. This adjustment resulted in the liquid biopsy panel detecting an additional three previously non-reported mutations (1 in UPS, 2 in GIST). LMS subjects were the most concordant of our STS subtypes.

### 2.4. Median Tumor Fraction Predicts Liquid/Solid Tumor CGP Concordance in STS

We next explored what factors may drive CGP concordance. Tumor subtype appears to be a key factor. LMS, as stated before, had the highest median estimated tumor fraction per cohort and the best concordance between liquid and solid tumor profiling (Figure 3B). We also note that there is a trend toward a linear correlation between tumor fraction and concordance among STS cohorts (R^2^ = 0.8, *p* = 0.09) (Figure 3C).

### 2.5. Liquid Tumor Profiling Demonstrates Limited Efficacy in the Detection of Copy Number Alterations and Losses

**Figure 4:**
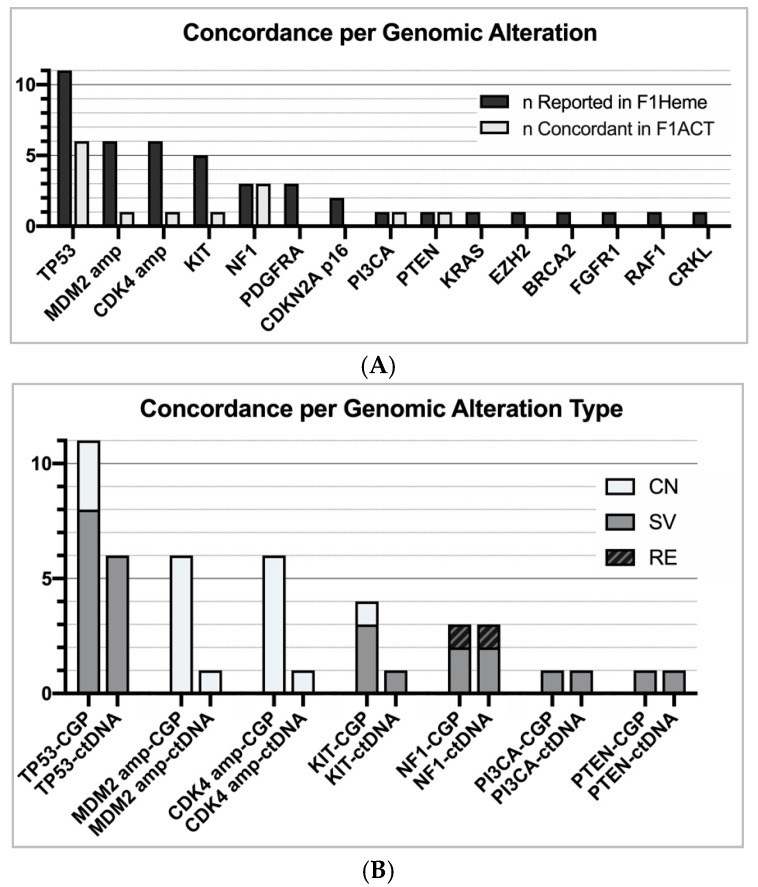
(**A**) Overall concordance between F1Heme and F1ACT per genomic alteration (see Figure S1 for genomic alterations reported in F1ACT not previously stated in F1Heme). (**B**) Concordance between liquid/solid CGP per genomic alteration type. F1ACT performs fairly well in the detection of short variant alterations (SV = 11/15). However, F1Heme is inadequate in the detection copy number alterations and losses (CN = 2/16).

In a further assessment of our data, we chose to analyze the overall concordance per genomic alteration, regardless of the subtype. Mutations in NF1 and TP53 were the most concordant (NF1 = 3/3, TP53 = 6/11). When exclusively examining short variant mutations, TP53 was detected in 6 of 8 subjects (Figure 4). Contrarily, mutations in CDKN2A, gene amplification, and losses were poorly recognized in F1ACT profiling (CDKN2A = 0/2, CDK4/MDM2 amplification = 2/12, loss = 0/4) (Figure 4). F1ACT does not currently report genomic losses. When non-shedding tumors (TF = 0) were excluded, the overall concordance increased from 35% to 42%. When only short variant alterations in shedding tumors were considered, 13 of 17 (76%) alterations were detected in the liquid biopsy with LMS having the highest concordance (5/5).

## 3. Discussion

The application of comprehensive genomic profiling has become a cornerstone in the treatment of various cancers. Newer ctDNA sequencing methods used in this study are pushing the forefront of disease detection, pathogenesis, and long-term monitoring. However, information regarding STS tumor shedding and the efficacy of these newer methods have heretofore yet to be reported in detail. Here we present our experiences prospectively evaluating 24 advanced STS subjects. We offer a robust analysis of the concordance between sequencing from two commercially available CLIA-certified solid tumor and ctDNA platforms to identify associated trends and obstacles.

It is clear that in an unselected population, ctDNA CGP in STS are poorly concordant with solid tumor CGP. Our data shows only 26% (7/24) of subjects with complete concordance and 46% (11/24) of subjects with complete/partial concordance. This is likely driven by the low estimated TF levels derived from blood from our subjects. Indeed, over 50% (13/24) subjects had a TF < 1%. Moreover, 6 of 13 registered had essentially undetectable TFs. This limitation stymied our initial hypothesis that STS intratumoral heterogeneity would provide an advantage to using ctDNA methods as they theoretically should provide a comprehensive, holistic mutational analyses. However, due to the low tumor fraction available in the blood we were unable to confirm these results.

Our data suggests that the amount of ctDNA shed into the blood is subtype specific. Interestingly, all LMS subjects had detectable TFs (average = 7.1% versus 0.48% in all other subtypes). This LMS TF percentage is in line with previously published data for its detectable nature [8]. As a point of reference, other cancers such as advanced metastatic breast tumors are reported to have an average TF of 4.5% (*n* = 80) and highly metastatic prostate tumors average a TF of 19% (*n* = 138) [13,14]. Future studies are needed to evaluate ctDNA profiling on a subtype-by-subtype basis.

A limitation of the study is that gene coverage of the F1ACT platform does not yet incorporate all sequenced exons on the solid tumor panel. In other words, F1ACT’s interrogation of 62 genes is difficult to compare alongside F1Heme’s over 400 genes and 250 RNA-sequencing platform. The tumor alteration status required to provide a clinician with actionable information is therefore not only limited by low TF levels observed in most STS but also with a limited number of genes. Newer liquid biopsies are being developed that will have additional genes (~300–500) which may obviate this weakness in the future. Another consideration when comparing the two tests is the presence of putative germline mutations. We categorized alterations as germline if the allele frequency was 50 ± 2% in both the tissue and blood testing. Using this definition, our study included 11 concordant germline mutations. The presence of germline mutations in ctDNA can significantly skew the estimated TF generated from the software. For example, one UPS subject reported a TF of 46.3% after liquid testing, but then later was determined to have two germline mutations. Because our analysis only attempts to consider tumor DNA shed from actual cancer and not all germline tissues, we chose to reevaluate TFs for subjects who reported germline mutations.

The study also highlights that current liquid biopsies techniques likely lack the dynamic range necessary for consistent detection of losses and copy number alterations. These classes of mutations are extremely difficult to detect in the blood using NGS methods due to high levels of baseline noise and is dependent on TF. In the technical specifications of F1ACT, detection of amplifications varies depending on amplitude of the CNA and ctDNA fraction; at TF > 20% the sensitivity of detection of CNA is approximately 95%. In our study, the TFs were significantly lower and therefore the sensitivity detecting CNAs was significantly lower. Therefore, it is not surprising that our results confirm this observation as only 2/16 of these alteration types were concordant between tests. If we were to include only cases with detectable TFs and assess only short variants, our concordance rate would increase from 35% (16/46) to 76% (13/17).

Finally, as mentioned prior the LMS cohort had the highest median TF and the highest concordance percentage. Thus, LMS subjects may potentially benefit from liquid biopsy protocols in future studies.

## 4. Materials and Methods

### 4.1. Sample Collection

We performed a prospective, single-institution biomarker trial (The Ohio State University IRB: OSU-17159) from June 2016 to November 2018 at The Ohio State University Sarcoma Clinic. Subjects over the age of 18 with an eligible sarcoma diagnosis were eligible for enrollment into the trial if they had measurable disease on CT or MRI imaging and had previously undergone FoundationOne Heme/Sarcoma (F1Heme) CGP. Subjects needed to have one of four groups of sarcoma diagnoses: DDLPS, LMS, UPS, or GIST. STS subjects within these cohorts with a radiographically measurable tumor were then approached for additional profiling using the FoundationACT™ assay (F1ACT). F1ACT evaluates rearrangements in 62 genes and targets ~141 kbp of the human genome including all exons of 27 genes, selected exons of an additional 34 genes (133 exons), selected introns of 6 genes frequently involved in genomic rearrangements in cancer (12 introns), and the TERT promoter region that is recurrently mutated in cancer, as previously described [11]. Of note, FoundationACT™ is now rebranded as the FoundationOne Liquid panel (Foundation Medicine, Cambridge, MA, USA).

### 4.2. Radiographic Analysis

Volumetric assessment of subject tumors was performed by a single radiologist using syngo.via (Siemens Healthcare, Malvern, PA, USA) and Agfa Impax PACS after importing CT scans performed at or around the time of F1ACT collection. Representative lesions were chosen in cases in which the presence of numerous lesions made determining the exact tumor volume difficult.

### 4.3. Tumor Fraction Determination

TF was determined by using the maximum somatic allele frequency (MSAF), a method of estimating the fraction of tumor-derived ctDNA versus that of total cell free DNA (cfDNA) and was calculated for all the samples. MSAF/TF was determined by calculating the allele fraction (AF) for all known somatic, likely somatic, and variants of unknown significance (VUS) substitution alterations detected on non-PCR-duplicate read pairs. Common and rare germline variants found in the ExAC database, dbSNP v135, and 1000 Genomes database were excluded from the MSAF calculation. A calculated MSAF value of 0 indicates that no ctDNA was detected in the sample.

### 4.4. Statistical Analysis

Genes and specific exons overlapping on both CGP panels were reviewed for subject-based concordance and alteration-specific concordance using descriptive statistics. We defined subject complete concordance describing all possible matching genes on both panels. Partial concordance was defined as at least one gene matching but not all possible genes. For this study, we chose to only report of the variants of known significance as arbitrated by ClinVar [12]. Alteration specific concordance for liquid biopsy was defined by the positive percent agreement (PPA). We define PPA as the number of alterations detected in the liquid biopsy divided by the number of alterations identified in the liquid and tissue biopsy. Correlation between circulating tumor fraction (TF) and tumor volume were evaluated using linear regression methods. Descriptive statistics and calculations were performed in Graphpad 8.0 Prism.

## 5. Conclusions

This report is the first study evaluating multiple subtypes of STS and their concordance between solid tumor CGP as compared to ctDNA analysis from blood. Aside from leiomyosarcoma, most sarcoma subtypes shed ctDNA at low fractions and serves as the limiting factor in both profiling effectiveness and the observed poor concordance with solid tumor results. STS tumor volume and metastatic burden are insufficient markers to suggest the presence ctDNA. Further, ctDNA platforms perform modestly in the detection of short variant alterations and may lack the necessary power to report copy number alterations and losses reliably. The modest concordance rates and median TF observed in LMS may justify the use of liquid biopsy panels for future studies.

## Figures and Tables

**Table 1:** Subject demographics. Twenty-seven subjects were enrolled—three subjects failed to complete testing.

Participant #	Sex	Age	Histological Subtype	Evidence of ctDNA
1	Female	73	Leiomyosarcoma	Yes
2	Female	91	Gastrointestinal stromal tumor	Yes
3	Female	53	Leiomyosarcoma	Yes
4	Male	76	Dedifferentiated liposarcoma	Yes
5	Female	68	Leiomyosarcoma	Yes
6	Female	67	Dedifferentiated liposarcoma	No
7	Female	55	Leiomyosarcoma	Yes
8	Male	73	Dedifferentiated liposarcoma	No
9	Male	74	Gastrointestinal stromal tumor	Yes
10	Male	82	Leiomyosarcoma	Yes
11	Female	57	Dedifferentiated liposarcoma	No
12	Male	67	Gastrointestinal stromal tumor	Yes
14	Male	47	Gastrointestinal stromal tumor	Yes
15	Male	81	Gastrointestinal stromal tumor	No
16	Female	62	Gastrointestinal stromal tumor	No
17	Female	64	Dedifferentiated liposarcoma	Yes
18	Female	69	Undifferentiated pleomorphic sarcoma	Yes
19	Female	36	Undifferentiated pleomorphic sarcoma	No
20	Female	68	Dedifferentiated liposarcoma	Yes
22	Male	75	Undifferentiated pleomorphic sarcoma	Yes
24	Male	71	Undifferentiated pleomorphic sarcoma	Yes
25	Male	60	Undifferentiated pleomorphic sarcoma	Yes
26	Female	64	Leiomyosarcoma	Yes
27	Male	81	Undifferentiated pleomorphic sarcoma	Yes

## Supplementary Materials

The following are available online at https://www.mdpi.com/2072-6694/11/12/1829/s1, Table S1: Summary of Clinical Characteristics, Figure S1: (A) No significant correlation between subjects’ TF and quantity of metastases. (B) nor tumor volume and quantity of metastases. (C) Tumor volumes across the four subtypes were comparable, Figure S2. FM Data Collection Form.
