# Supplementary material for: Prospective Evaluation of the Concordance of Commercial Circulating Tumor DNA Alterations with Tumor-Based Sequencing across Multiple Soft Tissue Sarcoma Subtypes
Source: Cancers (Basel). 2019 Nov 21;11(12):1829. doi: 10.3390/cancers11121829 (PMC6966562; doi:10.3390/cancers11121829)
Supplement: Supplementary file 1 [file cancers-11-01829-s001.pdf]

cancers

Supplementary Materials

Table S1. Summary of Clinical Characteristics.

Patient ID	Metastases	Tumor Fraction	Tumor Volume
1	5	0.2345	174.2
2	3	0.0157	69.3
3	3	0.0202	46.2
4	3	0.0019	75.7
5	2	0.1158	8069.5
6	5	0	294.7
7	2	0.0362	77.9
8	4	0	926.1
9	1	0.0014	4.5
10	6	0.0167	37.5
11	2	0	759
12	4	0.0036	127.6
14	5	0.013	179.9
15	1	0	79.7
16	2	0	3.6
17	4	0.0031	1019.8
18	5	0.0089	91.7
19	6	0	15.2
20	4	0.1086	1499.9
22	3	0.001	22.6
24	6	0.4623*	*1625.4*
25	4	0.0112	19.8
26	2	0.0022	3.5
27	1	0.0175	1608.4

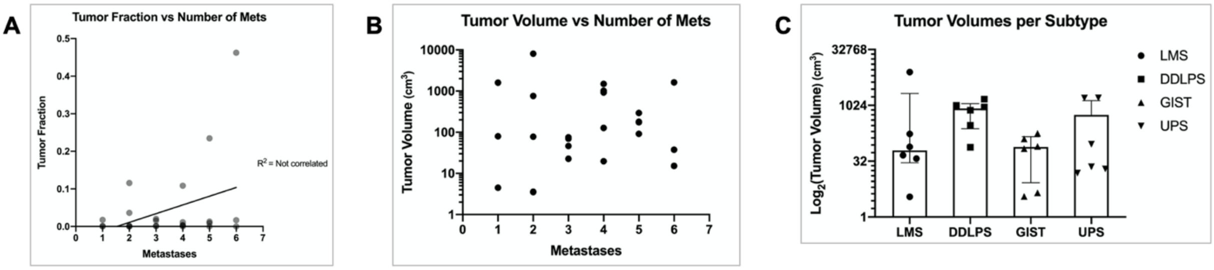

Figure S1. (**A**) No significant correlation between subjects' TF and quantity of metastases. (**B**) nor tumor volume and quantity of metastases. (**C**) Tumor volumes across the four subtypes were comparable

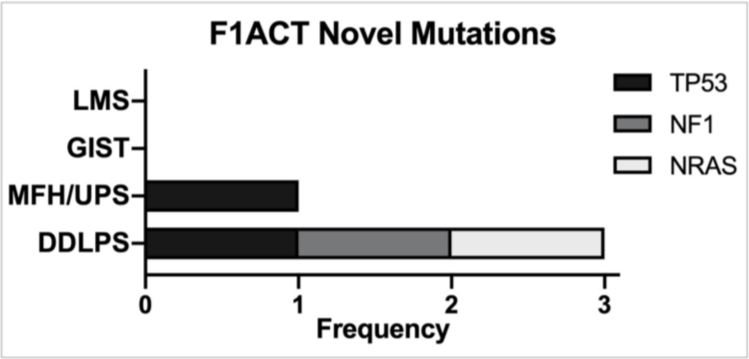

Figure S2. FM Data Collection Form. Across all 24 STS subjects who underwent both NGS assays, F1ACT methods identified four mutations not previously reported in F1Heme.
